# Effects of Prenatal Dexamethasone Treatment and Post-Weaning Moderate Fructose Intake on Synaptic Plasticity and Behavior in Adult Male Wistar Rat Offspring

**DOI:** 10.3390/biology13070547

**Published:** 2024-07-19

**Authors:** Đurđica Ignjatović, Nataša Nestorović, Mirko Tomić, Nataša Ristić, Nataša Veličković, Milka Perović, Milica Manojlović-Stojanoski

**Affiliations:** 1 Department of Biochemistry, Institute for Biological Research “Siniša Stanković”—National Institute of Republic of Serbia, University of Belgrade, 142 Despot Stefan Blvd., 11000 Belgrade, Serbia; mitomic@ibiss.bg.ac.rs (M.T.); nvelickovic@ibiss.bg.ac.rs (N.V.); 2 Department of Cytology, Institute for Biological Research “Siniša Stanković”—National Institute of Republic of Serbia, University of Belgrade, 142 Despot Stefan Blvd., 11000 Belgrade, Serbia; rnata@ibiss.bg.ac.rs (N.N.); negicn@ibiss.bg.ac.rs (N.R.); 3 Department of Neurobiology, Institute for Biological Research “Siniša Stanković”—National Institute of Republic of Serbia, University of Belgrade, 142 Despot Stefan Blvd., 11000 Belgrade, Serbia; milkap@ibiss.bg.ac.rs

**Keywords:** prenatal, dexamethasone, fructose, novel object recognition, hippocampus, glucocorticoid receptor, synaptic plasticity, programming

## Abstract

**Simple Summary:**

During pregnancy, maternal glucocorticoids control fetal growth and the maturation of fetal tissues. Synthetic glucocorticoids are commonly used to stimulate lung differentiation in pregnancies at high risk of premature birth. Despite their beneficial effects on fetal survival, their impact on the developing brain is less clear. Among postnatal factors that might have a profound effect on both the cognitive capacity and behavior of the offspring, high fructose consumption in the young population is of particular concern. The present study aimed to investigate the effects of prenatal synthetic glucocorticoid exposure additionally challenged with postnatal fructose overconsumption on locomotion, anxiety, and memory in adult male rat offspring. According to our results, prenatal glucocorticoid treatment induced changes in reactions to novel situations in male rats that might represent advantageous fetal developmental adaptation, while increased exploratory behavior, reduced anxiety, and improved ability to recognize novel objects could improve survival in an adverse postnatal environment. On the other hand, moderate fructose consumption did not appear to alter the effects of prenatal glucocorticoid exposure, suggesting that fetal programming had a predominant influence.

**Abstract:**

Early-life glucocorticoid overexposure induces diverse neurodevelopmental outcomes regarding stress reactivity and cognition. Increased fructose consumption has also been associated with alterations in cognitive capacity and behavior. The present study investigated the effects of prenatal dexamethasone exposure on synaptic plasticity, locomotion, anxiety, and recognition memory in adult male Wistar rat offspring, and whether these effects are potentiated by postnatal fructose consumption. Pregnant female rats were treated with dexamethasone during late gestation and male offspring were supplemented with a moderate dose of fructose. Recognition memory, locomotion, and anxiety-like behavior were assessed using a novel object recognition test, open-field test, and elevated plus maze, respectively. Hippocampal synaptic plasticity was estimated by the levels of growth-associated protein 43 (GAP-43), synaptophysin, postsynaptic density protein 95, calcium/calmodulin-dependent kinase IIα, and their activating phosphorylations. Additionally, protein levels of the glucocorticoid receptor (GR) and its transcriptionally active phosphorylated form were evaluated. Prenatal dexamethasone treatment induced an anxiolytic-like effect, stimulation of exploratory behavior, and novelty preference associated with an increase in GR and GAP-43 protein levels in the hippocampus. Fructose overconsumption after weaning did not modify the effects of prenatal glucocorticoid exposure. Applied prenatal dexamethasone treatment may induce changes in reactions to novel situations in male Wistar rats.

## 1. Introduction

Rapid growth and finely tuned development of the fetal brain during pregnancy make it particularly vulnerable to any hostile intra-uterine environment [1]. Alterations in conditions during critical stages of development lead to a set of permanent fetal adaptive changes, a process termed developmental programming. During prenatal development, glucocorticoids (GCs) control fetal growth, proliferation, and the maturation of numerous fetal tissues, including neuronal differentiation [2]. Maternal cortisol increases throughout normal gestation [3], providing sufficient cortisol levels during the third trimester, required for the maturation of the fetal organs and preparation for delivery [4]. In pregnant mothers under stress, high circulating GCs induce adaptive changes in fetal structure and metabolism that initially promote fetal survival, but are often associated with metabolic and mental health disorders later in life [5,6]. Besides endogenously derived hypercortisolism, prenatal treatments with synthetic corticosteroids in clinical practice have similar effects. Dexamethasone is a glucocorticoid receptor (GR) agonist commonly used to stimulate lung differentiation and newborn survival in pregnancies at high risk of preterm delivery [7]. However, it is well known that prenatal dexamethasone treatment also induces lower birth weight [8]. Several longitudinal studies have reported that prenatal exposure to synthetic corticosteroids is associated with greater distractibility and hyperactivity, neurosensory deficits, aggressive–destructive behavior, and higher emotionality in children [9,10,11,12]. Animal studies have also revealed that prenatal dexamethasone treatment induced increased stress reactivity [13], cognitive impairments associated with changes in hippocampal synaptic transmission [14], and a reduction in the number of proliferative cells in the hippocampus [15].

Among postnatal factors that might have a profound effect on both the metabolic and mental health of the offspring, high fructose consumption is particularly concerning in the young population [16,17]. Higher intake of fructose, mainly from beverage consumption, is a risk factor for the development of metabolic diseases [16]. Consequently, poor metabolic health is associated with the disruption of child cognitive development, even in healthy, typically developing children [18]. Recent studies have highlighted the role of the hippocampus in fructose-induced cognitive deficits, especially in sensitive periods of neurocognitive development—childhood and adolescence [19]. A high-fructose diet in adolescents elevates glucocorticoids and induces anxiety- and depressive-like behavior [17]. Moreover, high fructose consumption during infancy provokes different behavioral effects in male and female rats, with negative effects on attention and impulsivity noted only in males [20]. The focus of this study was to evaluate if fructose overconsumption during the childhood of male rats might affect possible behavioral responses induced by prenatal GC programming.

Fetal exposure to excessive glucocorticoids, natural or synthetic, also has sex-specific effects on offspring behavior. Males tend to show learning and memory deficits, while females show depressive-like and anxiety-like behavior [21]. Furthermore, male offspring exposed to maternal stress show stress-induced locomotor hyperactivity in adulthood [22]. In humans, prenatal glucocorticoid overexposure has also been associated with increased cortisol reactivity to acute psychosocial stress and depression in girls and increased risk of attention deficit hyperactivity disorder symptoms in boys [23,24,25]. 

The present study aimed to investigate the effect of dexamethasone prenatal treatment and postnatal moderate fructose consumption on recognition memory and general locomotion in Wistar Han male offspring. Additionally, to compare with our previously obtained results on females [26], anxiety-like behavior in males after identical treatment was estimated. Due to the hippocampal key role in the performance of recognition memory [27], hippocampal levels of proteins related to synaptic plasticity—growth-associated protein 43 (GAP-43), synaptophysin, postsynaptic density protein 95 (PSD-95) and its form phosphorylated at Serine 295—the ratios of total and auto-phosphorylated (Threonine 286) calcium/calmodulin-dependent kinase IIα (CaMKIIα) were estimated. Additionally, protein levels of the GR and its transcriptionally active form phosphorylated at Serine 232 (corresponding to human Serine 211), which is considered a biomarker for activated GR [28], were evaluated.

## 2. Materials and Methods

### 2.1. Animals and Treatment

**Figure 1:**
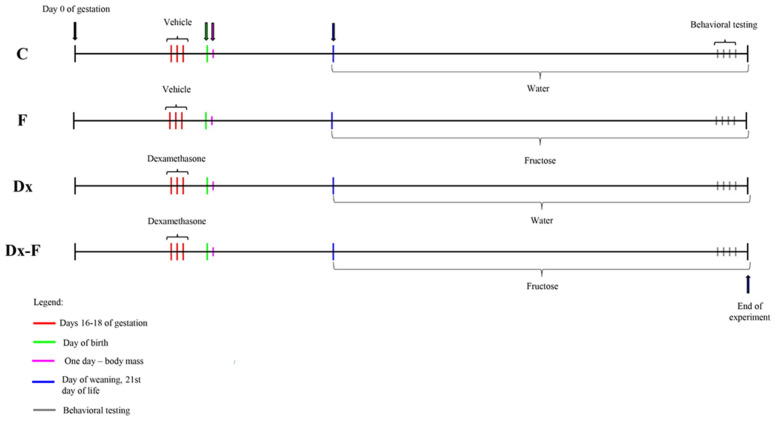
Experimental timeline.

Adult female Wistar Han rats (2–2.5 months old) were mated in the vivarium of the Institute for Biological Research, Belgrade, Serbia, during the night. Animals were maintained under standard conditions (23 ± 2 °C, 60–70% relative humidity, 12 h light/dark intervals), with food and water available ad libitum. In the morning, vaginal smears were analyzed, and sperm-positive vaginal smears were considered as an indication of pregnancy (day 0 of gestation). Gravid females were randomized into two groups. The experimental group received subcutaneous injection of dexamethasone (Dx) in a dose of 0.5 mg/kg/day on gestational days 16, 17, and 18, while control females were treated with the same quantity of saline. This particular dosing paradigm is well in line with the recommended range of clinical human exposure [7,29] and causes low birth weight in Wistar rats [30]. To check the effect of prenatal Dx overexposure, the body masses of one-day-old offspring of control and Dx-treated dams were measured. After weaning (21st day of life), to minimize litter effect, males were randomly chosen from control litters and litters of Dx-treated mothers, and divided into two more groups. The first group was fed with standard laboratory rodent chow (Veterinarski zavod Subotica, Serbia). Both food and drinking water were available ad libitum. The second group had ad libitum access to the same chow and 10% (*w*/*v*) fructose solution instead of drinking water. Thus, four groups were formed: control male offspring (C), male offspring supplemented with fructose in drinking water (F), male offspring from Dx-treated dams (Dx), and male offspring from Dx-treated dams supplemented with fructose in drinking water (Dx-F). Each group consisted of six males. The postnatal experimental procedure, which included water or 10% fructose consumption, lasted for 10 weeks. Male offspring were subjected to behavioral testing at the age of three months. After two days, animals were euthanized by rapid decapitation. The body mass was measured immediately before euthanasia. The experimental paradigm is presented as timeline (Figure 1).

All animal procedures complied with Directive 2010/ 63/EU on the protection of animals for experimental and other scientific purposes and the ethical standards of the Low Animal Welfare No 41/2009 as national guides on the care and use of laboratory animals, and were approved by the Ethical Committee for Use of Laboratory Animals of the Institute for Biological Research “Siniša Stanković”, University of Belgrade, No 7-12/12. The experiments were performed following a guideline on the principles of regulatory acceptance of 3Rs (replacement, reduction, refinement) testing approaches, European Medicines Agency, 2016.

### 2.2. Behavioral Tests

Animals were subjected to three behavioral tests in the adult period of life, i.e., at the age of three months, on four consecutive days between 9 A.M. and 1 P.M. It was supposed that the day-after-day submissions to tests might induce better habituation to stress in comparison to the interrupted exposure pattern [31]. Open-field test (OFT), performed on Day 1, is a standard assay for assessing locomotor activity in rodents by tracking their walking distance and periods of inactivity. Two other OFT parameters, the number of entries and the time spent in the central zone, were used to evaluate anxiety-like behavior, which was additionally investigated by elevated plus maze (EPM) on Day 4. Day 1 was also considered as the habitation phase for the Novel object recognition test (NOR), which was performed on Days 2 and 3. This test was used to measure behavior relevant to recognition memory. Both OFT and NOR were performed in the same apparatus, consisting of four adjacent plastic-coated open-field square areas (70 × 70 cm), enclosed and separated by plywood (H = 50 cm). The activity of up to four rats was synchronously and independently registered in this apparatus by a high-angle video camera, elevated at 2.40 m above ground level, and connected to a PC. The identically positioned camera was used to record rat behavior in EPM. All behavioral tests were performed in a separate dimly illuminated room (indirect 2 × 40 W light) with light and acoustic isolation, and the temperature was maintained at 25 °C. Video analysis was performed using ANY-maze software (ANY-maze Video Tracking System 4.30, Stoelting Co., Wood Dale, IL, USA). Particular behavioral parameters were identified by two proficient experimenters unaware of the experimental groups. After each test, the equipment was cleaned with 10% ethanol solution and dried with paper towels to remove any trace of odor.

### 2.3. Open-Field Test

Each animal was positioned in the center of the area, and its locomotion was recorded during the following 5 min interval. By ANY-maze analysis, two main parameters illustrating animal locomotor activity were defined: (1) total traveled distance (in meters), by tracking the center of the animal body; (2) time of inactivity (in seconds) as the sum of periods when animals did not express movement in space. Founded on the premise that rodents express anxious behavior in open fields by avoiding the central part of the area and prefer to move near the fence [32], the central region (35 × 35 cm), presenting ¼ of the total area, was observed to track rat ambulation. The time that the center of rat body spent in this region and the number of entrances during 5 min were analyzed and calculated to reflect their level of anxiety.

### 2.4. Novel Object Recognition Test

This test was established as a valuable measure of cognition and memory retention [33]. The variant of the NOR protocol applied in our experiments was adapted from the protocol [34]. Each animal was allowed a 10 min training session with exposure to two identical, non-toxic objects (hard plastic items) placed in the two opposite corners of the arena (70 × 70 cm). Following the training session, the animals were immediately returned to their home cage. After 24 h, each animal was returned to the same arena, in which one familiar object was replaced with a novel object of a similar size, but with a different shape and color. The animal was placed in the center of the arena facing away from both objects. The exploration was defined when the animal’s head was directed toward an object and inside a circle (R = 6 cm) around the object. The test was ended after accumulating 40 s of exploration time on either of the sample objects. The novel object preference ratios were calculated by dividing the novel object exploratory time by the time used to explore both objects (40 s). The sessions were video-recorded and subsequently analyzed by ANY-maze.

### 2.5. Elevated Plus Maze

Anxiety-like behavior was examined by EPM, according to the described procedure [35]. The EPM apparatus was made of blue acrylic and consisted of three zones: two opposite open arms (50 × 10 cm) and two opposite closed arms (50 × 10 cm) with 40 cm walls, connected by a central platform (10 × 10 cm). The cross-platform was elevated to a height of 60 cm. The testing was started by placing each rat in the central square of the maze facing one of the closed arms. Its behavior was recorded for the next 5 min with a video camera positioned vertically above the apparatus. Two basic parameters, the percentages of open arm entries and of time spent in open arms during 5 min, were calculated and analyzed. Additional ethological parameters [35], like the number of rearing, the time spent grooming, as well as behaviors related to risk assessment—head-dipping (exploratory scanning over the sides of the maze), closed-arm returns (c-returns) and stretched attend posture (SAP)—were also estimated. However, the results are presented only where there were significant effects of the treatments.

### 2.6. Serum Corticosterone Determination

Blood was collected from individual animals’ trunks in the morning hours, between 09:00 and 10:00 h, and the sera were stored at −80 °C until the analysis. Based on a previous pilot study, samples were diluted 20×, and total corticosterone, both bound and free, was determined using a commercially available ELISA kit, following manufacturer instructions (#KGE009, R&D Systems, Abingdon Science Park Abingdon, OX14 3NB, Abingdon, UK). Intra-assay and inter-assay coefficients of variations were 6.1% and 6.5%, respectively.

### 2.7. Preparation of Whole Cell Extract

After decapitation, brains were removed and hippocampi were dissected. To obtain whole-cell protein extracts, tissues were homogenized in 10 vol. (*w*/*v*) of ice-cold RIPA buffer (50 mM Tris, pH 7.5, 150 mM NaCl, 1% Nonidet P-40, 0.1% SDS, 0.5% Triton X-100, 1 mM EDTA, 1 mM EGTA, 2 mM DTT with protease and phosphatase inhibitors) using a glass/teflonhand homogenizer (Potter-Elvehjem, Deltalab, Barcelona, Spain). The homogenates were sonicated on ice (3 × 5 s at 10 MHz, Hielscher Ultrasound Processor, Hielscher Ultrasonics, Teltow, Germany), left for extraction for 30 min at 0 °C, and finally centrifuged at 20,000× *g* for 30 min. The resulting supernatants were stored at −70 °C. 

### 2.8. Western Blot Analysis

The concentration of isolated proteins was determined by the Lowry method, using bovine serum albumin (BSA) as a standard. Equal protein amounts (10 or 40 µg per lane) were separated by electrophoresis on 8% or 10% sodium dodecyl sulfate-polyacrylamide gels and transferred to polyvinylidene difluoride membranes (Immobilon-P, Merck Millipore Ltd., Tullagreen, Ireland). The membranes were blocked by 5% non-fat dry milk or 2% bovine serum albumin in phosphate-buffered saline (PBS, 1.5 mM KH2PO4, 6.5 mM Na2HPO4, 2.7 mM KCl, 0.14 M NaCl, pH 7.2) at room temperature for one hour, and then incubated overnight at 4 °C with following primary rabbit polyclonal antibodies: anti-PSD95 (#2507s, Cell Signaling; 1:1000), anti-phospho-PSD95-Ser295 (#45737s, Cell Signaling, Danvers, MA, USA; 1:1000), anti-phospho-GR-Ser211 (#4161s, Cell Signaling Massachusetts, USA; 1:1000), anti-CaMKIIα (sc-9035, Santa Cruz Biotechnology, Dallas, TX, USA; 1:6000), anti-phospho-CaMKIIα-Thr286 (sc-12886-R, Santa Cruz Biotechnology, Dallas, TX, USA; 1:6000), anti-GAP-43 (sc-10786, Santa Cruz Biotechnology, Dallas, TX, USA; 1:15,000), anti-β-actin (PA1-183, Thermo Fisher Scientific, Rockford, IL, USA; 1:2000) and anti-GAPDH (#2118, Cell Signaling, Massachusetts, USA; 1:10,000) used as equal loading controls. Anti-synaptophysin rabbit monoclonal (MA5-14532, Thermo Fisher Scientific, Rockford, IL, USA; 1:200) and anti-GR mouse monoclonal antibody (sc-393232, Santa Cruz Biotechnology, Dallas, TX, USA; 1:500) were also used. After extensive washing, membranes were incubated for 90 min with appropriate horseradish peroxidase-conjugated secondary antibody (#7074 or #7076 Cell Signaling, MA, USA; 1:2000). The immunoreactive protein bands were visualized by the chemiluminescence method using iBright FL1500 Imaging System, and quantitative analysis was performed using iBright Analysis Software V5.3.0 (Thermo Fisher Scientific, USA). 

The expression of the target proteins in each experimental group was determined as the fold change relative to the appropriate controls that were assigned the value 1 (n = 6).

### 2.9. Statistical Analyses

Physiological parameters, behavioral data, and Western blot (see Supplementary Materials) data are presented as mean ± standard error of the mean (SEM). Statistical analyses for the effects of fructose and Dx treatments were performed using two-way ANOVA (Prism 8, GraphPad Software Inc, San Diego, CA, USA). Further inter-group differences were evaluated by the Tukey post hoc test and were considered significant at *p* < 0.05. Effect sizes were calculated with partial eta squared coefficients ($\eta_{p}^{2}$) and interpreted as small effect ($\eta_{p}^{2}$ = 0.01), medium effect ($\eta_{p}^{2}$ = 0.06), and large effect ($\eta_{p}^{2}$ = 0.14).

## 3. Results

### 3.1. Physiological Parameters

The body mass of the one-day-old offspring was significantly reduced after dexamethasone (Dx) treatment of pregnant dams compared to the controls. The average body mass of the animals in the experimental groups did not differ significantly from the control group at the age of three months. Significant differences in circulating corticosterone concentration between the control and treated groups were not observed as well. All data are presented in Table 1.

### 3.2. Behavioral Testing

**Figure 2:**
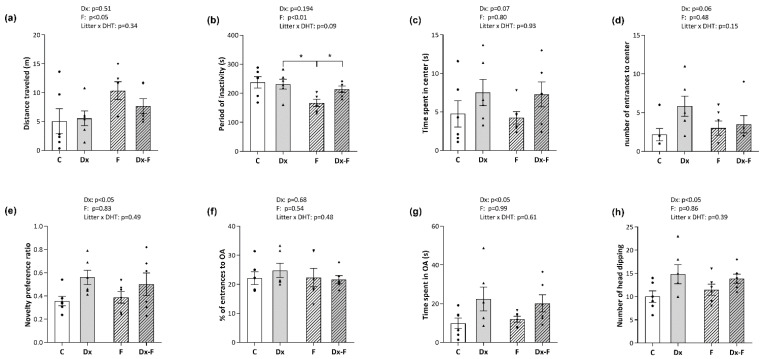
Animals were subjected to three behavioral tests, and parameters were measured and analyzed by ANY-maze software. Open-field test was used to evaluate (**a**) total locomotion by traveled distance and (**b**) time of inactivity during 5 min in the OF area. Two additional parameters, (**c**) the time spent in the central zone and (**d**) the number of entries to the center, were used to evaluate anxiety-like behavior. Cognition and memory retention were evaluated by a novel object recognition test and presented by the novel object preference ratios (**e**). Anxiety-like behavior was examined by Elevated Plus Maze during 5 min, where the parameters (**f**) percentage of the total entrances into open arms and (**g**) time spent in the open arms are presented. The number of head-dipping in 5 min (**h**) was identified and counted by the proficient experimenter. The data are presented as the mean ± SEM of n = 6 per group. Effects of fructose consumption and Dx treatment were determined by two-way ANOVA. A value of *p* < 0.05 was considered statistically significant. Significant between-group differences from post hoc Tukey test are given as * *p* < 0.05.

The effects of treatments on the animal locomotion were evaluated by open field (OF). The distance that rats traveled and the cumulative period of their inactivity during 5 min in the OF area were not affected by Dx treatment. However, two-way ANOVA revealed that fructose treatment affected both parameters. Namely, fructose-treated groups had longer traveled distances (F (1, 20) = 5.34, *p* < 0.05, $\eta_{p}^{2}$ = 0.21) and shorter periods of inactivity (F (1, 20) = 8.33, *p* < 0.01, $\eta_{p}^{2}$ = 0.29) (Figure 2a and Figure 2b, respectively). Post hoc analyses revealed that the period of inactivity was significantly decreased in the fructose group compared to the Dx group and Dx-F group (* *p* < 0.05, F vs. Dx or Dx-F).

The two parameters used for the assessment of anxiety-like behavior, the time spent (Figure 2c) and the number of entrances into the central quadrant of the area during 5 min (Figure 2d), were not significantly affected by the treatments. Still, an increase was noted for both parameters after Dx treatment near the level of significance (*p* = 0.07 and *p* = 0.06, respectively). Additionally, the data obtained for anxiety-like behavior tested by elevated plus maze (EPM) are presented in Figure 2f,g. The analyses did not show significant effects of the treatments on the percentage of entries in the open arms of the apparatus during 5 min (Figure 2f). Nevertheless, two-way ANOVA showed that Dx treatment increased the time spent in the open arms (F (1, 20) = 6.32, *p* < 0.05, $\eta_{p}^{2}$ = 0.24) (Figure 2g). Estimation of an array of registered ethological parameters in EPM revealed a stimulating effect of Dx treatment on head-dipping (F (1, 20) = 6.28, *p* < 0.05, $\eta_{p}^{2}$ = 0.24) (Figure 2h), while the other ethological parameters (i.e., number of rearings, time spent grooming, c-returns, and stretched attend posture (SAP)) were not affected by the treatments.

The novelty preference ratio obtained by novel object recognition (NOR) test, which reflects memory retention, was significantly elevated after Dx treatment (F (1, 20) = 5.71, *p* < 0.05, $\eta_{p}^{2}$ = 0.22) (Figure 2e).

### 3.3. Synaptic Plasticity Markers in the Hippocampus

**Figure 3:**
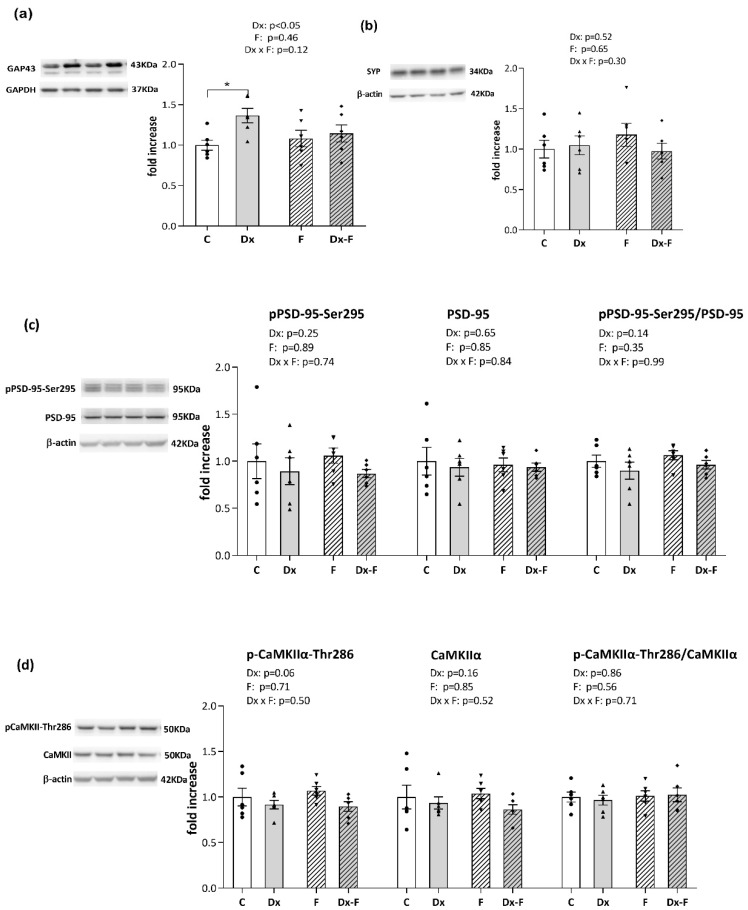
Representative Western blots and relative quantification of (**a**) GAP43, (**b**) synaptophysin, (**c**) pPSD-95-Ser295, total PSD, and their ratio, (**d**) pCAMKIIα-Thr286, total CAMKIIα, and their ratio, in the hippocampi of the prenatally Dx-treated and control rats, drinking water or fructose after weaning. β-actin and GAPDH were used for the normalization of immune-positive bands of target proteins. The expression of the target proteins in each experimental group was determined as the fold change relative to the appropriate controls that were assigned the value 1. The results are shown as mean ± SEM (n = 6 animals per group). Effects of fructose consumption and Dx treatment were determined by two-way ANOVA. A value of *p* < 0.05 was considered statistically significant. Significant between-group differences from post hoc Tukey test are given as * *p* < 0.05.

Recognized effects of Dx and fructose overconsumption on cognition prompted us to evaluate the expression of synaptic plasticity markers in our experimental paradigm. Protein levels of presynaptic markers, growth-associated protein 43 (GAP-43) and synaptophysin, together with the postsynaptic marker, postsynaptic density protein 95 (PSD-95) including its activating form phosphorylated at serine 295 (pPSD-95-Ser295), and the level of calcium/calmodulin-dependent kinase IIα (CaMKIIα) and its auto-phosphorylated form at threonine-286 (pCAMKIIα-Thr286) were analyzed in the hippocampi of the control and treated animals by Western blot analysis (Figure 3).

As shown in Figure 3a, two-way ANOVA revealed that Dx treatment increased the level of GAP-43 (F (1, 20) = 5.41, *p* < 0.05, $\eta_{p}^{2}$ = 0.21). Further post hoc analysis revealed that the protein level of GAP-43 was significantly increased in the Dx group compared to the control group (* *p* < 0.05, Dx vs. C).

Fructose and Dx treatments did not affect the level of synaptophysin, pPSD-95-Ser295, total PSD-95 or their ratio as well as p-CaMKIIα-Thr286, total CaMKIIα or their ratio (Figure 3b, Figure 3c and Figure 3d, respectively).

### 3.4. Glucocorticoid Receptor in the Hippocampus

**Figure 4:**
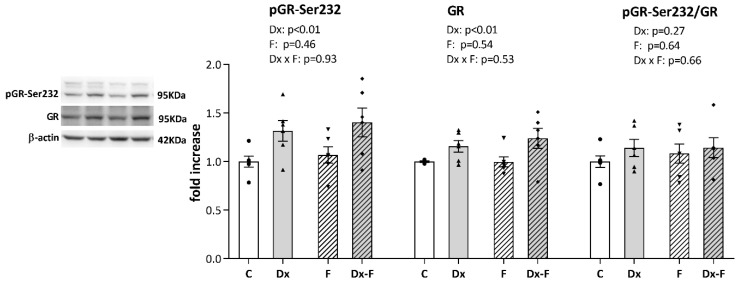
Representative Western blots and relative quantification of pGR-Ser232, total GR, and their ratio, in the hippocampi of the prenatally Dx-treated and control rats, drinking water or fructose after weaning. β-actin was used for the normalization of immune-positive bands of target proteins. The expression of the target proteins in each experimental group was determined as the fold change relative to the appropriate controls that were assigned the value 1. The results are shown as mean ± SEM (n = 6 animals per group). Effects of fructose consumption and Dx treatment were determined by two-way ANOVA. A value of *p* < 0.05 was considered statistically significant.

Since it is well known that signaling via glucocorticoid receptor (GR) modulates learning and memory processes, we further proceeded with Western blot analysis of GR and its stimulatory phosphorylation at Serine 232 (pGR-Ser232). As shown in Figure 4, a two-way ANOVA revealed that Dx treatment increased the total GR (F (1, 20) = 9.58, *p* < 0.01, $\eta_{p}^{2}$ = 0.32) and pGR-Ser232 (F (1, 20) = 9.90, *p* < 0.01, $\eta_{p}^{2}$ = 0.33) levels, while neither of the treatments affected the ratio of pGR-Ser232 to total GR. However, post hoc analysis did not show significant differences between groups, although the trends toward GR and pGR elevations in Dx-Fru animals vs. controls were observed (*p* = 0.07 and *p* = 0.06, respectively).

## 4. Discussion

This study evaluates the influence of Dx prenatal treatment and postnatal moderate fructose consumption on recognition memory performance, anxiety-like behavior and general locomotion in Wistar Han male offspring. Hippocampal synaptic plasticity and its glucocorticoid signaling are common targets for both glucocorticoids and high-fructose-mediated effects on cognition and behavior. Therefore, we investigated whether alterations in hippocampal synaptic plasticity and glucocorticoid signaling underlie recognition memory capacity. In that sense, Wistar Han rats were treated prenatally with clinically significant doses of the synthetic glucocorticoid Dx, and male offspring were additionally challenged by prolonged postnatal fructose consumption (~10% *w*/*v*). This experimental design originated from human and animal studies presenting the undesirable outcomes of the prenatal exposure to synthetic GCs (i.e., lower birth weight, adverse effects on offspring metabolism, neurodevelopment, cognition, and behavior) [36,37]. Among postnatal factors, increased fructose consumption during adolescence has also been associated with changes in cognitive capacity, behavior, and hippocampal structure and function [17,19,38]. Therefore, it can be assumed that fructose overconsumption might furthermore potentiate maladaptive behavioral responses induced by fetal programming. 

The effect of prenatal Dx treatment and fructose overconsumption after weaning on learning and memory was evaluated by the NOR test. Prenatal Dx treatment had a stimulatory effect on the novelty preference ratio in adult offspring (Figure 2e), indicating a positive effect of this treatment on recognition learning and memory capacity. A similar effect of prenatal Dx treatment on cognitive capacity was also found by Zeng et al., who reported that late gestational exposure of Wistar rats to Dx resulted in greater cognitive flexibility in male offspring [39]. The registered novelty preference in Dx groups was associated with increased levels of the synaptic plasticity marker GAP-43 in the hippocampus (Figure 3a), while the levels of synaptophysin, PSD-95, CaMKIIα, as well as their activatory phosphorylations (pPSD-95-Ser295 and pCAMKIIα-Thr286) were not altered. GAP-43 is involved in the regulation of presynaptic plasticity and memory formation [40], and previous studies have shown that moderate overexpression of this plasticity-associated protein can improve memory and regulate information storage [41,42]. While the stimulatory effect of a single high dose of Dx on GAP-43 levels in the hippocampus of aged rats [43] or of a Dx-releasing pellet on GAP-43 mRNA levels after peripheral nerve injury [44] is known, this is the first study showing this effect in prenatally Dx-exposed rats.

Exposure of the fetus to high levels of glucocorticoids, both exogenous and endogenous, can permanently affect GR expression [45]. Since glucocorticoid signaling is involved in the consolidation of contextual information, filtering, and integration of sensory stimuli [46], the novelty preference observed in our study may be related to upregulated hippocampal GR (Figure 4). Dose-dependent effects of glucocorticoids on memory consolidation were previously demonstrated. While moderate doses improve memory storage [47], lower and higher doses are less effective or even lead to memory impairment [48]. To that effect, elevated prenatal stress is associated with cognitive impairment [49,50], while mild prenatal stress can improve learning and reduce anxiety in offspring [51]. In addition to the dose, the timing of prenatal glucocorticoid exposure appears to be crucial for cognitive development. A well-designed longitudinal study in humans found that elevated cortisol concentrations at the beginning of pregnancy were associated with slower mental development, while elevated maternal cortisol concentrations at the end of pregnancy were associated with accelerated cognitive development [52]. Consequently, we believe that both the timing (late gestation) and dose (in the recommended clinical range) of prenatal Dx treatment in our study fall within the favorable range in the context of cognitive development.

Prenatal Dx treatment also induced an anxiolytic-like effect corresponding to increased time spent on open arms in EPM tests (Figure 2g). An analogous effect of Dx treatment detected by two parameters in OF (time spent in center and number of entrances to the center, Figure 2c and Figure 2d, respectively) was quite near the level of significance. This finding is consistent with the previously observed inhibition of anxiety-like behavior in Wistar–Kyoto rats by late gestational exposure to Dx [53]. Moreover, the number of head dippings as a measure of exploratory–risk assessment behavior in the EPM test [54] was also increased after prenatal Dx treatment (Figure 2h). A possible explanation is that prenatal Dx treatment in our experimental paradigm induced changes in reactions to novel situations in males that might be considered adaptive fetal programming, while increased exploratory behavior, reduced anxiety, and improved ability to recognize novel objects could improve survival in an adverse postnatal environment. However, increased exploratory behavior and lower anxiety may also increase risk of predation, making this phenotype strongly situation-dependent. On the other hand, in adult female offspring following identical prenatal Dx treatment, anxiety-like behavior was increased [26], which is in concordance with previously observed sex-specific differences in stress-related behavior after prenatal Dx exposure [55]. The behavioral changes observed in this study were not accompanied by variations in baseline corticosterone levels. This result is consistent with studies of other authors who reported that prenatal or neonatal treatment with synthetic steroids did not alter baseline corticosterone levels in offspring [26,56].

It must be noted that in the aforementioned papers, in which prenatal Dx treatment induced effects on behavior consistent with our results [39,53], gravid dams received a similar dosing regimen as ours during late gestation, designed to fall within the range of clinical human exposure [29]. Additionally, the same strain of Wistar rats were employed. Contrastingly, most of the adverse effects of the prenatal Dx treatment on cognitive functions and anxiety-like behavior were reported in Sprague Dawley rats [57,58]. These discrepancies might point out significance of strain selection in animal studies as well as caution when extrapolation of results from rodents to human is performed. In line with this, depending on dosage, time of exposure, sex, test paradigm, age, species, and strain employed, early glucocorticoid exposure in rodents results in contradictory cognitive capacity outcomes [57,59,60,61,62], as well as different behavioral responses [26,39,53,58,63]. 

Three months of ad libitum consumption of 10% (*w*/*v*) fructose solution in our study, from weaning to adulthood, which represents moderately increased fructose consumption mimicking unhealthy dietary habits [64], had no effect on body weight, serum corticosterone levels, anxiety-like behavior or novelty preference. The absence of the effect of moderate fructose consumption on body weight was not surprising, since studies investigating the effect of consumption of high concentrations of fructose report conflicting effects considering rodent body weight [65]. The only effect of fructose consumption in our study was increased locomotor activity. This is consistent with the observed slight increase in locomotor activity after chronic consumption of 10% fructose solution in young male Wistar rats [66] and early exposure to a high-fructose diet in Sprague Dawley rats [20]. However, our results are in contrast with previous studies that reported increased serum corticosterone and anxiety- and depression-like behaviors after consumption of a high-fructose diet (55% fructose) during adolescence in Wistar rats [17] or impaired recognition and spatial memory in Sprague Dawley rats after long-term intake of 10% fructose [50], or 30-day consumption of 11% high-fructose corn syrup [19]. These discrepancies suggest that the fructose dose and time regimen in our study were probably not strong enough to provoke changes in corticosterone levels and alterations in anxiety-like behavior or recognition memory. Since physiological outcomes associated with supraphysiological concentrations of fructose cannot be used to extrapolate the effects on human health, we have chosen to analyze the effect of lower-concentration fructose beverage consumption at concentrations similar to those found in sugar-sweetened beverages, which is reported to have adverse effects on metabolic health [65]. A recent paper revealed that the long-term consumption of 10% fructose during adolescence impaired spatial memory associated with neuroinflammation in male Wistar rats [67]; however, we did not find this effect of 10% fructose on recognition memory in our study. This discrepancy may be explained by the higher vulnerability of spatial memory to hippocampal dysfunction than recognition memory [27], implying that moderate fructose consumption does not affect less complex recognition memory but might harm more demanding spatial memory.

## 5. Conclusions

This study reveals that prenatal Dx treatment induces increased levels of synaptic plasticity marker GAP-43 and upregulation of GR in the hippocampus associated with novelty preference, improvements in exploratory behavior and certain anxiolytic-like effects. This implies that prenatal Dx treatment promotes changes in reactions to novel situations in male Wistar Han rats, which represent fetal developmental adaptation to a new environment. On the other hand, moderate fructose consumption after weaning did not affect any of the parameters analyzed in our experimental paradigm, except the stimulating effect on locomotion, suggesting that fetal programming had a prevailing influence.

## Figures and Tables

**Table 1:** Body mass and plasma corticosterone level.

Physiological Parameters	C	Dx	F	Dx-F
Body mass of one-day-old offspring (g)	6.58 ± 0.26	5.94 ± 0.29 *	/	/
Body mass of three-month-old offspring (g)	322.33 ± 22.18	318.33 ± 12.69	343.17 ± 14.97	351.00 ± 6.96
Corticosterone of three-month-old offspring (ng/mL)	44.56 ± 9.60	34.12 ± 7.32	46.94 ± 7.65	28.26 ± 3.18

All values are provided as the mean ± SEM; n = 12 for one-day-old offspring, n = 6 for three-month-old offspring. Four groups were formed after weaning: control male offspring (C), male offspring supplemented with fructose in drinking water (F), male offspring from Dx-treated dams (Dx), and male offspring from Dx-treated dams supplemented with fructose in drinking water (Dx-F). A value of *p* < 0.05 was considered statistically significant and is given * *p* < 0.05.

## Data Availability

The original contributions presented in the study are included in the article/Supplementary Material; further inquiries can be directed to the corresponding author.

## Supplementary Materials

The following supporting information can be downloaded at: https://www.mdpi.com/article/10.3390/biology13070547/s1. Western blot: Figure 2 Synaptic plasticity markers in the hippocampus; Figure 3 GR in the hippocampus.

## Abbreviations

GAP-43	growth-associated protein 43
PSD95	postsynaptic density protein 95
CAMKIIα	calcium/calmodulin-dependent kinase IIα
GR	glucocorticoid receptor
GCs	glucocorticoids
Dx	dexamethasone
C	control male offspring
F	male offspring supplemented with fructose in drinking water
Dx-F	male offspring from Dx-treated dams supplemented with fructose in drinking water
OFT	open-field test
EPM	elevated plus maze
NOR	novel object recognition test
SAP	stretched attend posture
BSA	bovine serum albumin
SEM	standard error of the mean

## References

[1] Fitzgerald E., Hor K., Drake A.J. (2020). Maternal influences on fetal brain development: The role of nutrition, infection and stress, and the potential for intergenerational consequences. Early Hum. Dev..

[2] Flagel S.B., Vazquez D.M., Watson S.J., Neal C.R. (2002). Effects of tapering neonatal dexamethasone on rat growth, neurodevelopment, and stress response. Am. J. Physiol. Regul. Integr. Comp Physiol..

[3] Sandman C.A., Glynn L., Schetter C.D., Wadhwa P., Garite T., Chicz-DeMet A., Hobel C. (2006). Elevated maternal cortisol early in pregnancy predicts third trimester levels of placental corticotropin releasing hormone (CRH): Priming the placental clock. Peptides.

[4] Hacking D., Watkins A., Fraser S., Wolfe R., Nolan T. (2001). Respiratory distress syndrome and antenatal corticosteroid treatment in premature twins. Arch. Dis. Child. Fetal Neonatal Ed..

[5] Fowden A.L., Vaughan O.R., Murray A.J., Forhead A.J. (2022). Metabolic Consequences of Glucocorticoid Exposure before Birth. Nutrients.

[6] Lewis A.J., Galbally M., Gannon T., Symeonides C. (2014). Early life programming as a target for prevention of child and adolescent mental disorders. BMC Med..

[7] Roberts D., Dalziel S. (2017). Antenatal corticosteroids for accelerating fetal lung maturation for women at risk of preterm birth. Cochrane Database Syst. Rev..

[8] Bloom S.L., Sheffield J.S., McIntire D.D., Leveno K.J. (2001). Antenatal dexamethasone and decreased birth weight. Obstet. Gynecol..

[9] French N.P., Hagan R., Evans S.F., Mullan A., Newnham J.P. (2004). Repeated antenatal corticosteroids: Effects on cerebral palsy and childhood behavior. Am. J. Obstet. Gynecol..

[10] Asztalos E., Willan A., Murphy K., Matthews S., Ohlsson A., Saigal S., Armson A., Kelly E., Delisle M.F., Gafni A. (2014). Association between gestational age at birth, antenatal corticosteroids, and outcomes at 5 years: Multiple courses of antenatal corticosteroids for preterm birth study at 5 years of age (MACS-5). BMC Pregnancy Childbirth.

[11] French N.P., Hagan R., Evans S., Godfrey M., Newnham J.P. (1998). Repeated Antenatal Corticosteroids (CS): Behaviour Outcomes in a Regional Population of Very Preterm (VP,<33w) Infants • 1252. Pediatr. Res..

[12] Trautman P.D., Meyer-Bahlburg H.F., Postelnek J., New M.I. (1995). Effects of early prenatal dexamethasone on the cognitive and behavioral development of young children: Results of a pilot study. Psychoneuroendocrinology.

[13] Hauser J., Feldon J., Pryce C.R. (2009). Direct and dam-mediated effects of prenatal dexamethasone on emotionality, cognition and HPA axis in adult Wistar rats. Horm. Behav..

[14] Luo M., Yi Y., Huang S., Dai S., Xie L., Liu K., Zhang S., Jiang T., Wang T., Yao B. (2023). Gestational dexamethasone exposure impacts hippocampal excitatory synaptic transmission and learning and memory function with transgenerational effects. Acta Pharm. Sin. B.

[15] Noorlander C.W., Tijsseling D., Hessel E.V., de Vries W.B., Derks J.B., Visser G.H., de Graan P.N. (2014). Antenatal glucocorticoid treatment affects hippocampal development in mice. PLoS ONE.

[16] Tappy L., Le K.A. (2010). Metabolic effects of fructose and the worldwide increase in obesity. Physiol. Rev..

[17] Harrell C.S., Burgado J., Kelly S.D., Johnson Z.P., Neigh G.N. (2015). High-fructose diet during periadolescent development increases depressive-like behavior and remodels the hypothalamic transcriptome in male rats. Psychoneuroendocrinology.

[18] Shapiro A.L.B., Wilkening G., Aalborg J., Ringham B.M., Glueck D.H., Tregellas J.R., Dabelea D. (2019). Childhood Metabolic Biomarkers Are Associated with Performance on Cognitive Tasks in Young Children. J. Pediatr..

[19] Hsu T.M., Konanur V.R., Taing L., Usui R., Kayser B.D., Goran M.I., Kanoski S.E. (2015). Effects of sucrose and high fructose corn syrup consumption on spatial memory function and hippocampal neuroinflammation in adolescent rats. Hippocampus.

[20] Barrett C.E., Jiang M., O’Flaherty B.G., Dias B.G., Rainnie D.G., Young L.J., Menigoz A. (2023). Early life exposure to high fructose diet induces metabolic dysregulation associated with sex-specific cognitive impairment in adolescent rats. J. Nutr. Biochem..

[21] Glover V., Hill J. (2012). Sex differences in the programming effects of prenatal stress on psychopathology and stress responses: An evolutionary perspective. Physiol. Behav..

[22] Bronson S.L., Bale T.L. (2014). Prenatal stress-induced increases in placental inflammation and offspring hyperactivity are male-specific and ameliorated by maternal antiinflammatory treatment. Endocrinology.

[23] Van den Bergh B.R., Van Calster B., Smits T., Van Huffel S., Lagae L. (2008). Antenatal maternal anxiety is related to HPA-axis dysregulation and self-reported depressive symptoms in adolescence: A prospective study on the fetal origins of depressed mood. Neuropsychopharmacology.

[24] Alexander N., Rosenlocher F., Stalder T., Linke J., Distler W., Morgner J., Kirschbaum C. (2012). Impact of antenatal synthetic glucocorticoid exposure on endocrine stress reactivity in term-born children. J. Clin. Endocrinol. Metab..

[25] Li J., Olsen J., Vestergaard M., Obel C. (2010). Attention-deficit/hyperactivity disorder in the offspring following prenatal maternal bereavement: A nationwide follow-up study in Denmark. Eur. Child. Adolesc. Psychiatry.

[26] Manojlović-Stojanoski M., Nestorović N., Petković B., Balind S.R., Ristić N., Trifunović S., Ajdžanović V., Filipović B., Šošić-Jurjević B., Milošević V. (2020). The effects of prenatal dexamethasone exposure and fructose challenge on pituitary-adrenocortical activity and anxiety-like behavior in female offspring. Tissue Cell.

[27] Broadbent N.J., Squire L.R., Clark R.E. (2004). Spatial memory, recognition memory, and the hippocampus. Proc. Natl. Acad. Sci. USA.

[28] Wang Z., Frederick J., Garabedian M.J. (2002). Deciphering the phosphorylation “code” of the glucocorticoid receptor in vivo. J. Biol. Chem..

[29] Carbone D.L., Zuloaga D.G., Hiroi R., Foradori C.D., Legare M.E., Handa R.J. (2012). Prenatal dexamethasone exposure potentiates diet-induced hepatosteatosis and decreases plasma IGF-I in a sex-specific fashion. Endocrinology.

[30] Ristić N., Nestorović N., Manojlovi-Stojanoski M., Trifunović S., Ajdžanović V., Filipović B., Pendovski L., Milošević V. (2021). Prenatal dexamethasone exposure and developmental programming of the ovary of the offspring: A structural study in the rat. Reprod. Fertil. Dev..

[31] Zhang W., Hetzel A., Shah B., Atchley D., Blume S.R., Padival M.A., Rosenkranz J.A. (2014). Greater physiological and behavioral effects of interrupted stress pattern compared to daily restraint stress in rats. PLoS ONE.

[32] Blanchard R.J., Blanchard D.C. (1989). Attack and defense in rodents as ethoexperimental models for the study of emotion. Prog. Neuropsychopharmacol. Biol. Psychiatry.

[33] Antunes M., Biala G. (2012). The novel object recognition memory: Neurobiology, test procedure, and its modifications. Cogn. Process..

[34] Hammond R.S., Tull L.E., Stackman R.W. (2004). On the delay-dependent involvement of the hippocampus in object recognition memory. Neurobiol. Learn. Mem..

[35] Rodgers R.J., Cao B.J., Dalvi A., Holmes A. (1997). Animal models of anxiety: An ethological perspective. Braz. J. Med. Biol. Res. = Rev. Bras. Pesqui. Medicas Biol..

[36] Aghajafari F., Murphy K., Matthews S., Ohlsson A., Amankwah K., Hannah M. (2002). Repeated doses of antenatal corticosteroids in animals: A systematic review. Am. J. Obstet. Gynecol..

[37] Fee E.L., Stock S.J., Kemp M.W. (2023). Antenatal steroids: Benefits, risks, and new insights. J. Endocrinol..

[38] Clark K.A., Alves J.M., Jones S., Yunker A.G., Luo S., Cabeen R.P., Angelo B., Xiang A.H., Page K.A. (2020). Dietary Fructose Intake and Hippocampal Structure and Connectivity during Childhood. Nutrients.

[39] Zeng Y., Brydges N.M., Wood E.R., Drake A.J., Hall J. (2015). Prenatal glucocorticoid exposure in rats: Programming effects on stress reactivity and cognition in adult offspring. Stress.

[40] Holahan M.R. (2017). A Shift from a Pivotal to Supporting Role for the Growth-Associated Protein (GAP-43) in the Coordination of Axonal Structural and Functional Plasticity. Front. Cell. Neurosci..

[41] Holahan M.R., Honegger K.S., Tabatadze N., Routtenberg A. (2007). GAP-43 gene expression regulates information storage. Learn. Mem..

[42] Routtenberg A., Cantallops I., Zaffuto S., Serrano P., Namgung U. (2000). Enhanced learning after genetic overexpression of a brain growth protein. Proc. Natl. Acad. Sci. USA.

[43] Tesic V., Perovic M., Zaletel I., Jovanovic M., Puskas N., Ruzdijic S., Kanazir S. (2017). A single high dose of dexamethasone increases GAP-43 and synaptophysin in the hippocampus of aged rats. Exp. Gerontol..

[44] Yao G.L., Kiyama H. (1995). Dexamethasone enhances level of GAP-43 mRNA after nerve injury and facilitates re-projection of the hypoglossal nerve. Brain Res. Mol. Brain Res..

[45] Levitt N.S., Lindsay R.S., Holmes M.C., Seckl J.R. (1996). Dexamethasone in the last week of pregnancy attenuates hippocampal glucocorticoid receptor gene expression and elevates blood pressure in the adult offspring in the rat. Neuroendocrinology.

[46] Korte S.M. (2001). Corticosteroids in relation to fear, anxiety and psychopathology. Neurosci. Biobehav. Rev..

[47] Sandi C. (1998). The role and mechanisms of action of glucocorticoid involvement in memory storage. Neural Plast..

[48] Andreano J.M., Cahill L. (2006). Glucocorticoid release and memory consolidation in men and women. Psychol. Sci..

[49] Fu Y., Liu H., He L., Ma S., Chen X., Wang K., Zhao F., Qi F., Guan S., Liu Z. (2022). Prenatal chronic stress impairs the learning and memory ability via inhibition of the NO/cGMP/PKG pathway in the Hippocampus of offspring. Behav. Brain Res..

[50] Wu Y., Espinosa K.M., Barnett S.D., Kapse A., Quistorff J.L., Lopez C., Andescavage N., Pradhan S., Lu Y.C., Kapse K. (2022). Association of Elevated Maternal Psychological Distress, Altered Fetal Brain, and Offspring Cognitive and Social-Emotional Outcomes at 18 Months. JAMA Netw. Open.

[51] Fujioka T., Fujioka A., Tan N., Chowdhury G.M., Mouri H., Sakata Y., Nakamura S. (2001). Mild prenatal stress enhances learning performance in the non-adopted rat offspring. Neuroscience.

[52] Davis E.P., Sandman C.A. (2010). The timing of prenatal exposure to maternal cortisol and psychosocial stress is associated with human infant cognitive development. Child. Dev..

[53] Lalonde C., Grandbois J., Khurana S., Murray A., Tharmalingam S., Tai T.C. (2021). Late gestational exposure to dexamethasone and fetal programming of abnormal behavior in Wistar Kyoto rats. Brain Behav..

[54] Hoshino K., Uga D.A., de Paula H.M. (2004). The compulsive-like aspect of the head dipping emission in rats with chronic electrolytic lesion in the area of the median raphe nucleus. Braz. J. Med. Biol. Res. = Rev. Bras. Pesqui. Medicas Biol..

[55] Hiroi R., Carbone D.L., Zuloaga D.G., Bimonte-Nelson H.A., Handa R.J. (2016). Sex-dependent programming effects of prenatal glucocorticoid treatment on the developing serotonin system and stress-related behaviors in adulthood. Neuroscience.

[56] Kamphuis P.J., Bakker J.M., Broekhoven M.H., Kunne C., Croiset G., Lentjes E.G., Tilders F.J., van Bel F., Wiegant V.M. (2002). Enhanced glucocorticoid feedback inhibition of hypothalamo-pituitary-adrenal responses to stress in adult rats neonatally treated with dexamethasone. Neuroendocrinology.

[57] Zheng Y., Zhang Y.M., Tang Z.S., Du J.K., Guo D.W., Xu Y.J., Sheng H., Lu J.Q., Ni X. (2021). Spatial learning and memory deficits induced by prenatal glucocorticoid exposure depend on hippocampal CRHR1 and CXCL5 signaling in rats. J. Neuroinflamm..

[58] Nagano M., Ozawa H., Suzuki H. (2008). Prenatal dexamethasone exposure affects anxiety-like behaviour and neuroendocrine systems in an age-dependent manner. Neurosci. Res..

[59] Yates N.J., Robertson D., Rodger J., Martin-Iverson M.T. (2016). Effects of Neonatal Dexamethasone Exposure on Adult Neuropsychiatric Traits in Rats. PLoS ONE.

[60] Wang Y.C., Huang C.C., Hsu K.S. (2010). The role of growth retardation in lasting effects of neonatal dexamethasone treatment on hippocampal synaptic function. PLoS ONE.

[61] Tsai K.J., Sze C.I., Lin Y.C., Lin Y.J., Hsieh T.H., Lin C.H. (2016). A Single Postnatal Dose of Dexamethasone Enhances Memory of Rat Pups Later in Life. PLoS ONE.

[62] Kreider M.L., Levin E.D., Seidler F.J., Slotkin T.A. (2005). Gestational dexamethasone treatment elicits sex-dependent alterations in locomotor activity, reward-based memory and hippocampal cholinergic function in adolescent and adult rats. Neuropsychopharmacology.

[63] Tsiarli M.A., Rudine A., Kendall N., Pratt M.O., Krall R., Thiels E., DeFranco D.B., Monaghan A.P. (2017). Antenatal dexamethasone exposure differentially affects distinct cortical neural progenitor cells and triggers long-term changes in murine cerebral architecture and behavior. Transl. Psychiatry.

[64] Kovacevic S., Nestorov J., Matic G., Elakovic I. (2014). Dietary fructose-related adiposity and glucocorticoid receptor function in visceral adipose tissue of female rats. Eur. J. Nutr..

[65] Toop C.R., Gentili S. (2016). Fructose Beverage Consumption Induces a Metabolic Syndrome Phenotype in the Rat: A Systematic Review and Meta-Analysis. Nutrients.

[66] Franco-Perez J., Manjarrez-Marmolejo J., Ballesteros-Zebadua P., Neri-Santos A., Montes S., Suarez-Rivera N., Hernandez-Ceron M., Perez-Koldenkova V. (2018). Chronic Consumption of Fructose Induces Behavioral Alterations by Increasing Orexin and Dopamine Levels in the Rat Brain. Nutrients.

[67] Fierros-Campuzano J., Ballesteros-Zebadua P., Manjarrez-Marmolejo J., Aguilera P., Mendez-Diaz M., Prospero-Garcia O., Franco-Perez J. (2022). Irreversible hippocampal changes induced by high fructose diet in rats. Nutr. Neurosci..

