# Supplementary material for: Effects of Prenatal Dexamethasone Treatment and Post-Weaning Moderate Fructose Intake on Synaptic Plasticity and Behavior in Adult Male Wistar Rat Offspring
Source: Biology (Basel). 2024 Jul 19;13(7):547. doi: 10.3390/biology13070547 (PMC11274266; doi:10.3390/biology13070547)
Supplement: Supplementary file 1 [file biology-13-00547-s001.zip › biology-3072502-supplementary.pdf]

ECL iBright Western Blot Imaging Systems Thermo Fisher

All experiments were performed in triplicates.
All experimental samples and controls used for one comparative analysis were run on the same blot/gel.

IC—internal control,
Molecular weight—Mw—according to Prestained Protein Ladder, Thermo Scinetific #26619 (10 to 250 kDa)

Figure 2 Synaptic plasticity markers in the hippocampus

GAP43

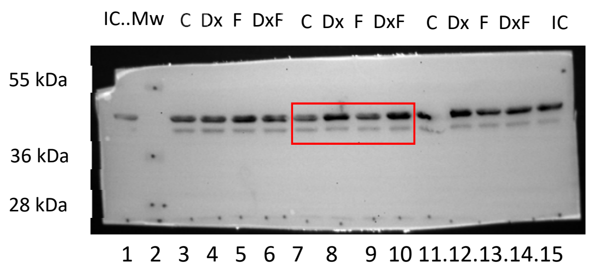

GAPDH for GAP43

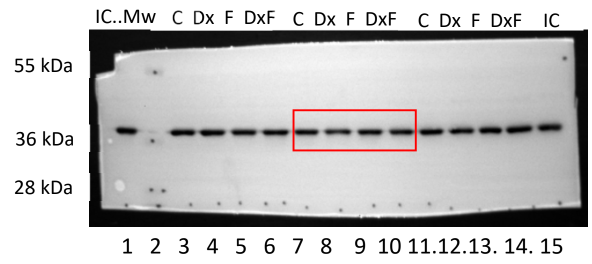

SYP

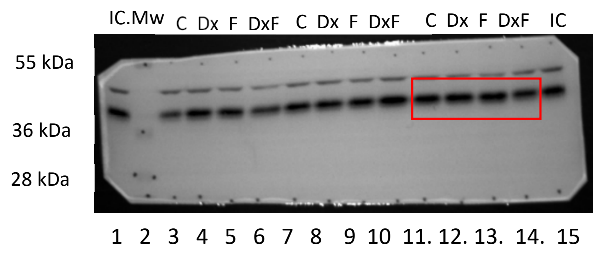

β-actin for SYP

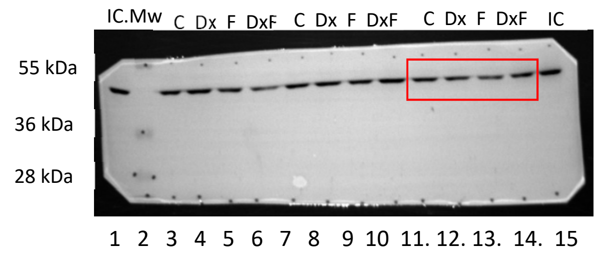

pPSD-95-Ser295	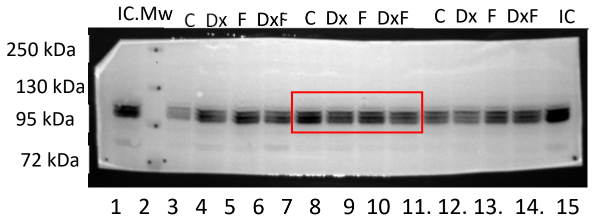
PSD-95	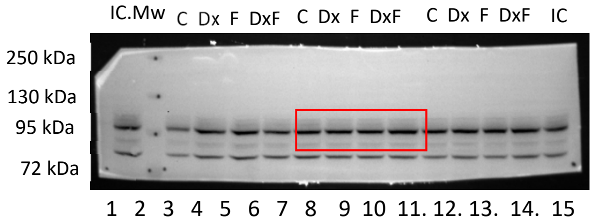
β-actin for PSD-95	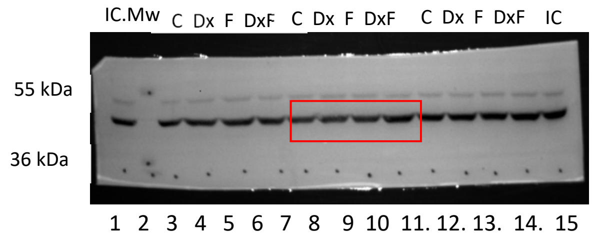
pCAMKII-Thr286	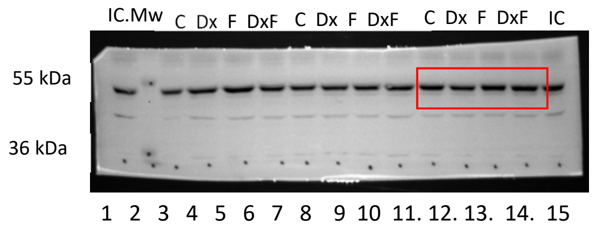
CAMKII	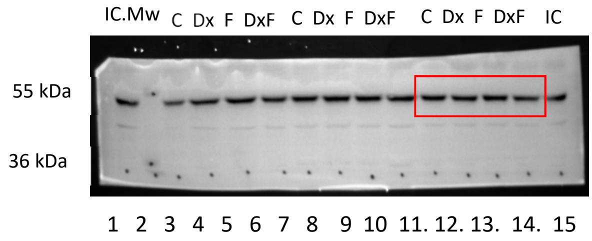
β-actin for CAMKII	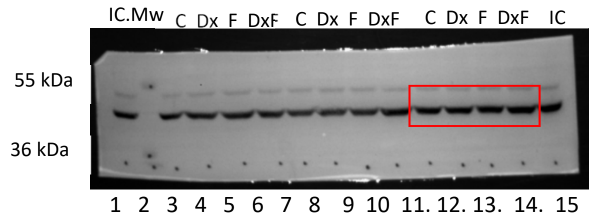

**Figure 3 GR in the hippocampus**	
pGR	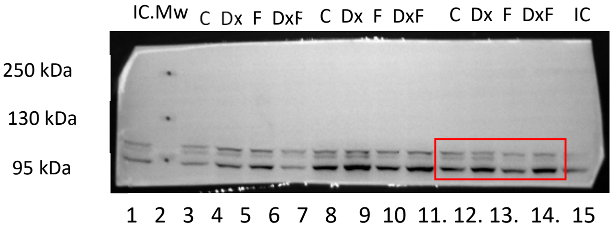
GR	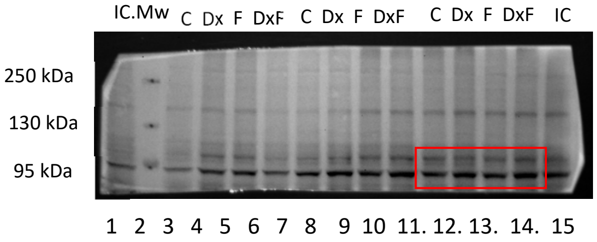
actin	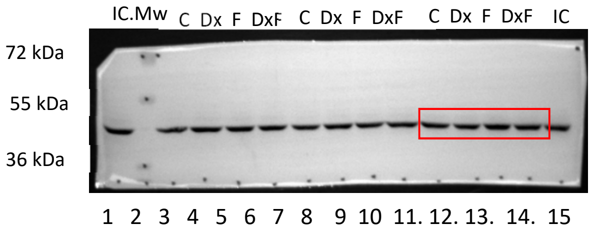
